# Adaptive Unscented Kalman Filter Phase Unwrapping Method and Its Application on Gaofen-3 Interferometric SAR Data

**DOI:** 10.3390/s18061793

**Published:** 2018-06-02

**Authors:** Yandong Gao, Shubi Zhang, Tao Li, Qianfu Chen, Shijin Li, Pengfei Meng

**Affiliations:** 1 School of Environment Science and Spatial Informatics, China University of Miningand Technology, Xuzhou 221116, China; ydgao@cumt.edu.cn (Y.G.); zhangsbi@163.com (S.Z.); Shijin_Li@cumt.edu.cn (S.L.); mpfcumt@163.com (P.M.); 2 Satellite Surveying and Mapping Application Center, National Administration of Surveying, Mapping and Geo-Information, Haidian District, Beijing 100048, China; chenqf@sasmac.cn

**Keywords:** phase unwrapping, Gaofen-3 SAR, AUKF, circular median filter, robustness

## Abstract

Phase unwrapping (PU) is a key step in the reconstruction of digital elevation models (DEMs) and the monitoring of surface deformation from interferometric synthetic aperture radar (SAR, InSAR) data. In this paper, an improved PU method that combines an amended matrix pencil model, an adaptive unscented kalman filter (AUKF), an efficient quality-guided strategy based on heapsort, and a circular median filter is proposed. PU theory and the existing UKFPU method are covered. Then, the improved method is presented with emphasis on the AUKF and the circular median filter. AUKF has been well used in other fields, but it is for the first time applied to interferometric images PU, to the best of our knowledge. First, the amended matrix pencil model is used to estimate the phase gradient. Then, an AUKF model is used to unwrap the interferometric phase based on an efficient quality-guided strategy based on heapsort. Finally, the key results are obtained by filtering the results using a circular median. The proposed method is compared with the minimum cost network flow (MCF), statistical cost network flow (SNAPHU), regularized phase tracking technique (RPTPU), and UKFPU methods using two sets of simulated data and two sets of experimental GF-3 SAR data. The improved method is shown to yield the greatest accuracy in the interferometric phase maps compared to the methods considered in this paper. Furthermore, the improved method is shown to be the most robust to noise and is thus most suitable for PU of GF-3 SAR data in high-noise and low-coherence regions.

## 1. Introduction

Interferometric synthetic aperture radar (SAR, InSAR) is one of the main methods for generating digital elevation models (DEMs) and monitoring terrain deformation [1,2]. In recent years, many countries have developed their own SAR satellites. On 10 August 2016, China launched the Gaofen-3 (GF-3) SAR sensor at the Taiyuan Satellite Launch Center. The GF-3 SAR satellite is a civilian microwave-based imaging satellite, China’s first C-band SAR satellite. With 12 imaging modes, it is the SAR satellite with the most number of imaging modes in the world [3,4]. The resolution of GF-3 image is 1~500 m [5]. This sensor has the advantages of high precision SAR internal calibration technology, high attitude control accuracy and good stability [6]. Several interferograms are obtained by controling the recursive baseline as short as possible. However, the coherence is not high given that the revisite time of GF-3 SAR satellite is nearly a month. The temporal coherence lowers the enssemble coherence. A more robust phase unwrapping (PU) method is required in GF-3 InSAR data processing [7,8]. 

In recent years, a number of new PU methods have been proposed [8,9,10]. Those can be divided into two categories [11,12,13]. The first one, called path-tracking [14,15], is represented by the branch-cut, the region-growing, the minimum discontinuity, and the minimum cost network flow (MCF) methods [16]. These methods select the appropriate PU starting point using a quality map, where the differences in the values of adjacent pixels are defined as phase gradients. The phase gradients are integrated to yield the unwrapped phase. While these methods have a higher unwrapped efficiency, the phase gradients are based on the quality of adjacent pixels, and hence these methods are highly dependent on adjacent pixels. The second category of PU methods, called minimum norm, is represented by the least squares (L^2^-norm) method [17,18] and involves global optimization. They transform the PU into the best unwrapped result in terms of the L^P^-norm. In 2000, Chen and Zebker proposed the famous statistical cost network flow (SNAPHU) method based on statistical models [17]. These methods have the advantages of requiring minimal computation, but they lead to the overall degradation of the unwrapped result due to the global error propagation. Since the interferogram is affected by noise, both of the above categories of PU methods need to be filtered before the PU [19]. The pre-filtering will directly affect the accuracy of PU [20], especially in high-noise and low-coherence regions.

Traditional PU methods require pre-filtering. Excessive filtering leads to loss of phase information, while insufficient filtering leads to residual error. In recent years, a PU method with simultaneous filtering was proposed. In 1999 and 2002, Manuel Servin and Juan Antonio Quiroga et al. proposed a PU method using regularized phase tracking technique (RPTPU) that can perform simultaneous noise filtering and PU [21,22]. Even in high noise areas, good results can still be obtained. However, RPTPU requires iterative calculation, which increases the amount of calculation and reduces the computational efficiency. In 1999, Kim and Griffiths first proposed that kalman filtering can be used for PU [23]. In 2008, Loffeld and Nies et al. proposed an extended kalman filter PU method [9,24]. However, these methods are based on two adjacent pixels along the vertical or horizontal axes. Hence, these methods will yield a greater PU error and possibly even PU failure. In 2009, Osmanoǧlu addressed the above problem and proposed a new extended kalman filter (EKF) PU method [25]. However, this method only considers the first-order nonlinearity of the phase, which will cause loss of phase information. To solve this problem, in 2011, Xie and Pi presented a new PU method based on an unscented kalman filter (UKF) with a path-tracking strategy and an omni-directional local phase gradient estimator [26]. In 2014, Xie and Li presented an enhanced PU method by combining an unscented kalman filter, an enhanced local phase gradient estimator based on an amended matrix pencil model, and a path-tracking strategy [27]. In 2015, Liu and Bian et al. first applied cubature kalman filter (CKF) to the PU of D-InSAR [13]. In 2016 and 2017, Xie proposed two improved UKFPU methods by combining the UKF with an amended matrix pencil model and an efficient quality-guided strategy. While these methods have demonstrated improvements in efficiency and accuracy of PU [28,29], the robustness of PU methods is yet to be improved, especially in high-noise and low-coherence regions.

In this paper, an improved UKFPU method is proposed. The proposed method, called AUKFMPU, combines the amended matrix pencil model, an adaptive UKF (AUKF), a quality-guided strategy based on heapsort, and circular median filtering to effectively increase the robustness of UKFPU in high-noise and low-coherence regions. This method also works well in high-noise and low-coherence regions. The paper also uses two sets of simulation data and two sets of GF-3 SAR experimental data to validate the proposed approach and compares it with MCF, SNAPHU, RPTPU, and UKFPU. The results show that the improved method in this paper has better robustness than other methods. It is proved that the method is more suitable than the other methods for PU of GF-3 SAR data. 

The paper is organized as follows. In Section 2, PU theory and the UKFPU method are introduced. In Section 3, the adaptive UKF and circular median filter components of the proposed PU method are outlined. In Section 4, simulation and experimental results and presented with discussions. The summary and conclusions are provided in Section 5.

## 2. Phase Unwrapping Theory and UKFPU Algorithms

### 2.1. Phase Unwrapping Theory

PU is a processing step used to estimate the true phase from the wrapped phase [30,31]. The complex interferometric phase is obtained by conjugating the two SAR images [32,33] with

(1) $$\varphi (k)=a(k)\exp j(\tilde{\phi }(k))$$

where φ(k) is the complex interferometric phase after conjugation multiplication, a(k) is the interferometric amplitude, and $\tilde{\phi }(k)$ is the modulo-2π interferometric phase. The measured complex interferometric phase has the following relationship with the true interferometric phase:

(2) $$\tilde{\phi }(k)={\left|\phi (k)+\omega (k)\right|}_{2\pi }=\phi (k)+\tilde{\omega }(k)\pm 2n\pi \in \left(-\pi ,\pi \right]$$

where ϕ(k) is the true unambiguous phase, ω(k) is the true phase error, and $\tilde{\omega }(k)$ is the mapped phase error. According to Equation (2), the difference between two adjacent pixels is

(3) $$\Delta_{\phi }(k)=\tilde{\phi }(k+1)-\tilde{\phi }(k)$$

Equation (2) into the Equation (3) can be obtained.

(4) $$\Delta_{\phi }(k)={\left|\phi (k+1)-\phi (k)+\omega (k+1)-\omega (k)\right|}_{2\pi }={\left|\delta (k)+\omega (k+1)-\omega (k)\right|}_{2\pi }$$

where δ(k) is the complex interferometric phase gradient and it is generally less than π. It can be seen from Equation (3) that when the phase has no noise, the true phase gradients can be obtained by determining the complex interferometric phase gradients [33]. So, the true phase of adjacent pixels can be expressed as

(5) $$\phi (k+1)=\phi (k)+\Delta_{\phi }(k)$$

However, noise in the phase is unavoidable, so PU cannot be performed by estimating the gradient using Equation (3). Interferometric phase filtering is usually required before PU. The quality of the pre-filtering, which will directly affect the PU quality, severely limits the application of the traditional PU methods. 

### 2.2. UKFPU Algorithms

x(k) represents value of the k pixel in the interferometric phase map, and the one-dimensional coordinate is replaced by the two-dimensional coordinate (m,n); that is, it is converted into a two-dimensional PU. According to the relationship between the interferometric phases of adjacent pixels and taking the real and imaginary components of the normalized complex interferometric phases as the two observations of the interferometric phase [13,26],

(6) x(k+1)=x(k)+u(k)+w(k)=f[x(k)]+w(k)y(k+1)={IM[z(k)]a(k)Re[z(k)]a(k)}={sin(x(k+1))cos(x(k+1))}+{v1(k+1)v2(k+1)}=h[x(k+1)]+v(k+1)

where u(k) is the true phase gradient at pixel k and w(k) is the phase gradient estimation error, assumed to be Gaussian white noise. z(k) is the complex interferometric phase value of the k pixel, a(k) is the amplitude of the k pixel, and $v_{1}(k)$ and $v_{1}(k)$ are the variance of the observations, assumed to be Gaussian white noise. For the state variable x, its Sigma point can be expressed as

(7) $$\begin{array}{l} \xi_{0}=\bar{x}(k),\\ \xi_{i}=\bar{x}(k)+{\left\{\sqrt{\left(n_{x}+\lambda )\bar{P}(k\right)}\right\}}_{i}\\ \xi_{i}=\bar{x}(k)-{\left\{\sqrt{\left(n_{x}+\lambda )\bar{P}(k\right)}\right\}}_{i} \end{array}$$

where $\bar{P}(k)$ and ${\left\{\sqrt{\left(n_{x}+\lambda )\bar{P}(k\right)}\right\}}_{i}$ represent the estimation error covariance matrix of the state variable x and the *i*-th vector of the $\left(n_{x}+\lambda )\bar{P}(k\right)$ root-mean-square matrix respectively, with the following weight coefficients:

(8) $$\begin{array}{l} b_{i}^{m}=\eta /(N+\eta )\;i=1,2,\cdots ,N\\ b_{i}^{c}=\eta /(N+\eta )+(1-\omega^{2}+l)\;i=0\\ b_{i}^{m}=b_{i}^{c}=1/2(N+\eta )\;i=1,2,\cdots ,2N \end{array}$$

where N represents the dimension of the state variable x (i.e., N=1); $\eta =\omega^{2}(N+\kappa )-N$, ω, l, κ are parameters used to adjust the Sigma point. In this paper, ω=0.01, l=2, κ=0. Assuming that the state estimates of phase k pixels in the phase diagram of the interferogram and their estimation error covariances are $\vec{x}(k)$ and $P_{xx}(k)$, respectively, the prediction formula is

(9) $$\begin{array}{l} \chi_{i}^{-}(k+1)=f\left[\chi_{i}(k)\right]\\ {\vec{x}}^{-}(k+1)=\sum_{i=0}^{2N}b_{i}^{m}\chi_{i}^{-}(k+1)\\ P_{xx}^{-}(k+1)=\sum_{i=0}^{2N}b_{i}^{c}\left[\chi_{i}^{-}(k+1)-{\vec{x}}^{-}(k+1)\right]\cdot [\chi_{i}^{-}(k+1)-{\vec{x}}^{-}(k+1){]}^{T}+Q(k) \end{array}$$

where $\chi_{i}^{-}(k+1)$ and ${\vec{x}}^{-}(k+1)$ represent the predicted value of the Sigma point of the k+1 pixel and the predicted value of the state variable of the k+1 pixel, respectively, Q(k) and $P_{xx}^{-}(k+1)$ represent the variance of the phase error of the k pixel and the covariance of the prediction error of the k+1 pixel prediction. So, its update variance can be expressed as

(10) $$\xi_{i}^{-}(k+1)=h\left[\chi_{i}^{-}(k+1)\right]$$

(11) $${\vec{y}}^{-}(k+1)=\sum_{i=0}^{2N}b_{i}^{m}\xi_{i}^{-}(k+1)$$

(12) $$P_{yy}^{-}(k+1)=\sum_{i=0}^{2N}b_{i}^{c}\left[\xi_{i}^{-}(k+1)-{\vec{y}}^{-}(k+1)\right]\cdot [\xi_{i}^{-}(k+1)-{\vec{y}}^{-}(k+1){]}^{T}+R(k+1)$$

(13) $$P_{xy}^{-}(k+1)=\sum_{i=0}^{2N}b_{i}^{c}\left[\chi_{i}^{-}(k+1)-{\vec{x}}^{-}(k+1)\right]\cdot [\xi_{i}^{-}(k+1)-{\vec{y}}^{-}(k+1){]}^{T}$$

(14) $$\tilde{\omega }(k+1)=P_{xy}^{-}(k+1)/P_{yy}^{-}(k+1)$$

(15) $$\vec{x}(k+1)={\vec{x}}^{-}(k+1)+\tilde{\omega }(k+1)\cdot \left[y(k+1)-{\vec{y}}^{-}(k+1)\right]$$

(16) $$P_{xx}(k+1)=P_{xx}^{-}(k+1)-\tilde{\omega }(k+1)\cdot P_{xy}^{-}(k+1)\cdot \tilde{\omega }{\left(k+1\right)}^{T}$$

In Equations (10)–(16), $P_{xx}^{-}(k+1)$ represents the covariance of the prediction of the k+1 pixel, y(k+1) and ${\vec{y}}^{-}(k+1)$ are the actual and the predicted value of the interferogram k+1 pixel observation value, respectively, $\tilde{\omega }(k+1)$ is the gain matrix of the k+1 pixel, R(k+1) represents the variance of the measurement error of the k+1 pixel, and $\vec{x}(k+1)$ and $P_{xx}(k+1)$, respectively, represent the variance of the estimation error of the k+1 pixel state estimate of the interferometric pattern. 

Equations (7)–(16) represent one-dimensional UKFPU models. The unwrapped pixels in the two-dimensional unwrapping are obtained by optimizing the weighted values of the unwrapped pixels in the eight neighboring pixels. Replace (m,n) with k in the one-dimensional model so that (16) is rewritten as

(17) $$\begin{array}{l} \chi_{i}^{-}(m,n)=\sum_{\left(a,s)\in (G\cap L\right)}d(a,s)\chi_{i}^{-}\left[\left(m,n)|(a,s\right)\right]\\ {\vec{x}}^{-}(m,n)=\sum_{i=0}^{2N}b_{i}^{m}\chi_{i}^{-}(m,n)\\ P_{xx}^{-}(m,n)=\sum_{i=0}^{2N}b_{i}^{c}\left[\chi_{i}^{-}(m,n)-{\vec{x}}^{-}(m,n)\right]\cdot [\chi_{i}^{-}(m,n)-{\vec{x}}^{-}(m,n){]}^{T}+\sum_{\left(a,s)\in (G\cap L\right)}d(a,s)Q(a,s) \end{array}$$

(18) $$d(a,s)=\frac{{\left[P_{xx}(a,s)\times \frac{1}{SNR(a,s)}\right]}^{-1}\cdot g(a,s)}{\sum_{\left(a,s)\in (G\cap L\right)}{\left[P_{xx}(a,s)\times \frac{1}{SNR(a,s)}\right]}^{-1}\cdot g(a,s)}$$

## 3. The Improved UKFPU Method

An improved UKFPU that combines an amended matrix pencil model, an AUKF, a quality-guided strategy based on heapsort, and a circular median filtering is proposed in this paper. This method applies the adaptive UKF to the PU for the first time.

### 3.1. The Adaptive UKF

As the conventional UKFPU method adopts a relatively fixed state and observation equation, it is difficult to control the stability of the model when the error varies greatly [34]. In order to reduce the influence of model error, this paper adds an adaptive factor to the UKFPU model to increase the robustness of the model as follows:

(19) $${\bar{P}}_{yy}^{-}(k+1)=\frac{1}{\alpha_{k}}\sum_{i=0}^{2N}b_{i}^{c}\left[\xi_{i}^{-}(k+1)-{\vec{y}}^{-}(k+1)\right]\cdot [\xi_{i}^{-}(k+1)-{\vec{y}}^{-}(k+1){]}^{T}+R(k+1)$$

(20) $${\bar{P}}_{xy}^{-}(k+1)=\frac{1}{\alpha_{k}}\sum_{i=0}^{2N}b_{i}^{c}\left[\chi_{i}^{-}(k+1)-{\vec{x}}^{-}(k+1)\right]\cdot [\xi_{i}^{-}(k+1)-{\vec{y}}^{-}(k+1){]}^{T}$$

(21) $$\bar{\tilde{\omega }}(k+1)=P_{xy}^{-}(k+1)/P_{yy}^{-}(k+1)$$

(22) $$\vec{x}(k+1)={\vec{x}}^{-}(k+1)+\bar{\tilde{\omega }}(k+1)\cdot \left[y(k+1)-{\vec{y}}^{-}(k+1)\right]$$

(23) $${\bar{P}}_{xx}(k+1)=\frac{1}{\alpha_{k}}P_{xx}^{-}(k+1)-\bar{\tilde{\omega }}(k+1)\cdot P_{xy}^{-}(k+1)\cdot \tilde{\omega }{\left(k+1\right)}^{T}$$

where $\alpha_{k}$ is the adaptive factor; ${\bar{P}}_{yy}^{-}(k+1)$, ${\bar{P}}_{xy}^{-}(k+1)$, $\bar{\tilde{\omega }}(k+1)$, $\vec{x}(k+1)$, and ${\bar{P}}_{xx}(k+1)$ are the covariances and final updated values obtained after adding the adaptive factor. Four methods for determining the value of $\alpha_{k}$ were proposed in the literature [35]. In this paper, by predicting the residual statistics, the adaptive factor can be calculated according to the following third-order formula:

(24) $$\alpha_{k}=\{\begin{array}{c} 1\\ \frac{c_{0}}{\left|\Delta {\bar{V}}_{k}\right|}{\left(\frac{c_{1}-\left|\Delta {\bar{V}}_{k}\right|}{c_{1}-c_{0}}\right)}^{2}\\ 0 \end{array}\;\begin{array}{cc} & \left|\Delta {\bar{V}}_{k}\right|\leq c_{0}\\ c_{0}< & \left|\Delta {\bar{V}}_{k}\right|\leq c_{1}\\ & \left|\Delta {\bar{V}}_{k}\right|>c_{1} \end{array}$$

where $\Delta {\bar{V}}_{k}$ denotes the learning statistic based on the predicted residual, $\Delta {\bar{V}}_{k}={\left(\frac{{\bar{V}}_{k}^{T}{\bar{V}}_{k}}{tr(P_{{\bar{V}}_{k}})}\right)}^{\frac{1}{2}}$; $P_{{\bar{V}}_{k}}=H_{k}P_{xx}^{-}(k+1)H_{k}^{T}+R(k+1)$, ${\bar{V}}_{k}$ is the update vector; $1.0\leq c_{0}\leq 1.5$ and $3.0\leq c_{1}\leq 8.5$. In Equation (24), $\alpha_{k}\neq 0$, when Equation (22) is used, the adaptive factor based on the two-segment function should be adopted as

(25) $$\alpha_{k}=\{\begin{array}{c} \begin{array}{c} 1\\ \frac{c_{0}}{\left|\Delta {\bar{V}}_{k}\right|} \end{array} \end{array}\;\begin{array}{c} \left|\Delta {\bar{V}}_{k}\right|\leq c\\ \left|\Delta {\bar{V}}_{k}\right|>c \end{array}$$

In general, 1.0≤c≤2.5 and the optimal value of *c* is 1.0. Equations (19)–(24) are the improved UKF data formula used in this paper.

### 3.2. The Circular Median Filter

The circular median filter shows a prominent advantage in detail retention ability. At the same time, the result of median filtering does not depend on the noise distribution model, so it is more adaptable to noise [36]. The circular median filter is given by

(26) $$\phi_{out}(l,m)=median\left[\arg\left[\frac{Q_{kl,km}}{d_{l,m}}\right]\right]+\arg(d_{l,m})$$

where $\phi_{out}(l,m)$ is the filter output signal; median[⋅] represents the middle of all the elements in the window centered on (l,m); arg[⋅] is the difference of matrix vector and the main vector in point (l,m) in the window, $d_{l,m}$ is the sum of the vectors in the filter window, and $Q_{kl,km}$ is the main vector in the window. Therefore, the circular median filter may be expressed as

(27) $$d_{l,m}=\sum_{kl=l-L_{D}}^{l+L_{D}}\sum_{km=m-L_{D}}^{m+L_{D}}Q_{kl,km}$$

This paper, for the first time, combines the AUKF with the circular median filtering to achieve PU. This improved method has higher robustness, especially in high-noise regions. 

## 4. Results and Discussion

Two simulated datasets and two sets of experimental GF-3 SAR data are processed by the MCF, SNAPHU, RPTPU, UKFPU, and AUKFMPU methods. The results are presented and analyzed in this section. The simulated datasets are obtained using MATLAB, while the parameters of the two sets of experimental GF-3 SAR data are given in Table 1.

### 4.1. Robustness Analysis

**Figure 1:**
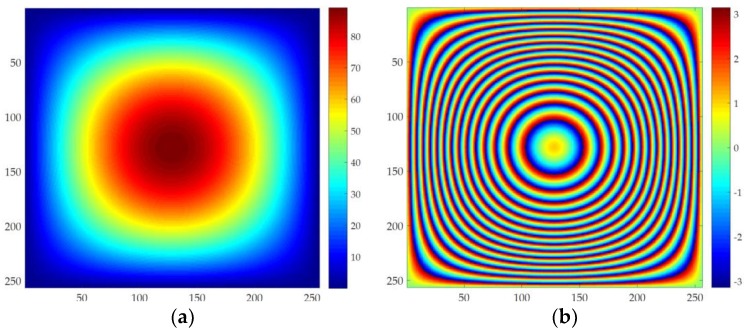
The simulated data used for robustness analysis. (**a**) is the simulated unwrapped phase; (**b**) is the corresponding wrapped phase.

As shown in Figure 1, in order to reduce the impact of other errors on different methods. We analyze the robustness of different methods using simulated data with sparse fringe. In order to verify the robustness of the proposed method, different levels of noise are added to the simulated data in Figure 1, with SNR of 9 dB, 5 dB, 3 dB, 1 dB, 0.8 dB, 0.5 dB, 0.3 dB, and 0.2 dB. Five different PU methods, i.e., MCF, SNAPHU, RPTPU, UKFPU, and AUKFMPU, are used to unwrap the simulated data with noise, and the robustness of different methods is compared. We use root mean square error (RMSE) to evaluate the PU accuracy of different methods. MCF method is implemented in Gamma software. SNAPHU is implemented in Doris software, while the RPTPU, UKFPU, and AUKFMPU methods are implemented using private code.

**Figure 2:**
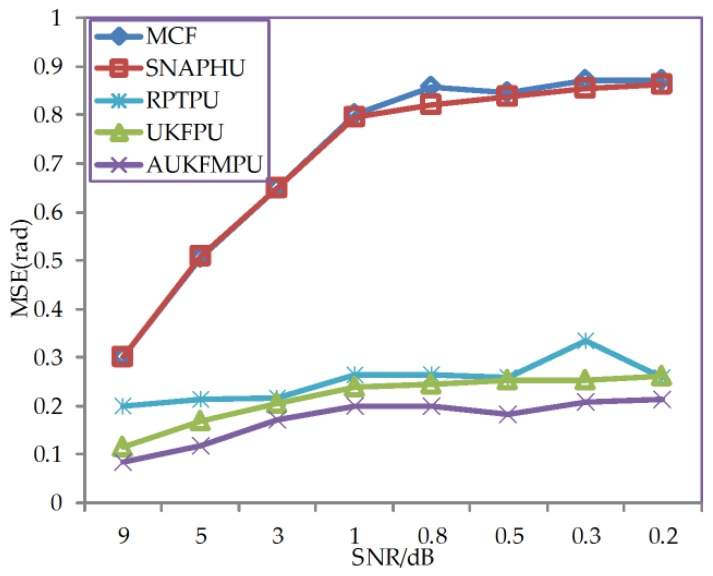
The line chart of RMSE against SNR for MCF, SNAPHU, RPTPU, UKFPU, and AUKFMPU methods.

From Table 2 and Figure 2, it can be seen that the RMSE of MCF and SNAPHU methods increases significantly with the decrease of the SNR. The RPTPU method has better robustness than the former two methods. And the PU result of RPTPU is very similar to UKFPU. Both methods can get good results when the noise is large, but the robustness still needs to be improved. It can be seen that the improved method proposed in this paper yields the lowest RMSE even when the SNR is low. Thus, the improved method proposed in this paper offers better robustness against noise than the other methods and is more suitable for PU in high-noise regions. The RPTPU, UKFPU, and AUKFMPU experiments in this letter run on a personal computer with Core i7-6700HQ CPU and MATLAB (R2016b). The size of simulation data is 256 × 256. The RPTPU consumes 182.5310 s to process the data, while the UKFPU and AUKFMPU use 2.410 s and 3.1494 s, respectively. Considering the robustness and efficiency of different methods, we can see that the advantages of AUKFMPU proposed in this paper are more obvious than other methods. According to the experiment, it can be found that the PU accuracy of the RPTPU is similar to the UKFPU. However, UKFPU is more robust than RPTPU, and UKFPU is much more efficiency than RPTPU. Therefore, the following experiments in this paper we only compare MCF, SNAPHU, UKFPU, and AUKFMPU.

### 4.2. Simulated Dataset 2 Results

**Figure 3:**
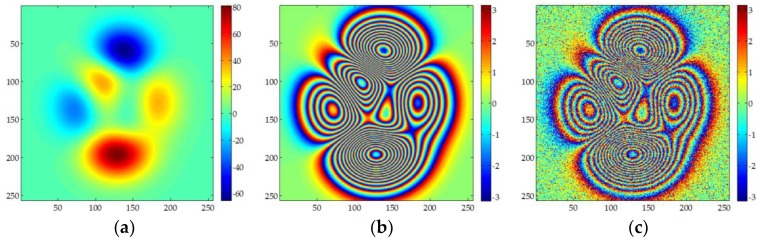
Simulated dataset 2: (**a**) is the simulated unwrapped phase; (**b**) is the corresponding wrapped phase; (**c**) is the data calculated by adding noise into (**b**), the noise RMSE is 0.65 radian.

Figure 3 shows the simulated data used in this section. The size of the data is 256 × 256 pixels. The RMSE quantifies the added noise. MCF, sSNAPHU, UKFPU, and AUKFMPU are used to process the simulated data respectively, and the unwrapping accuracy of these methods is compared. MCF method is implemented in Gamma software, SNAPHU is implemented in Doris software, while the UKFPU and AUKFMPU methods are implemented using private code. The ADF filter in Gamma software is used to filter the simulated data. The FFT window uses 32 × 32, and the threshold is 0.25.

**Figure 4:**
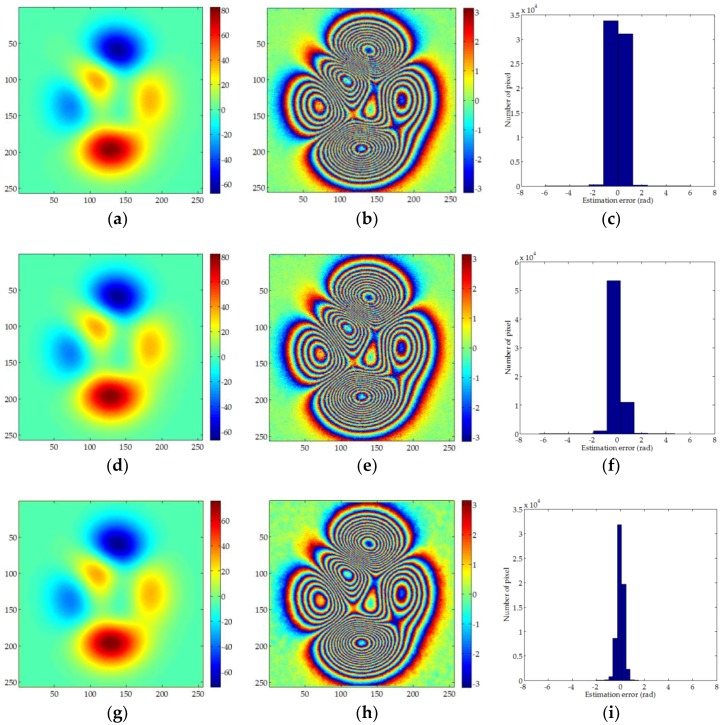

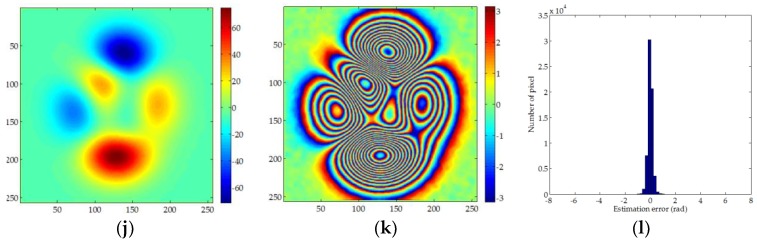
Unwrapped maps, re-wrapped maps and estimation error histogram for: (**a**–**c**) MCF; (**d**–**f**) SNAPHU; (**g**–**i**) UKFPU; and (**j**–**l**) AUKFMPU methods.

As shown in Figure 4, a–c are the PU results, re-wrapped interferometric phase, and estimation error of MCF, respectively. It can be seen that the MCF can get a better unwrapping effect after filtering. However, from the re-wrapped data we can see that some noise still remains. Figure 4d–f are the results of SNAPHU, and it can be seen that the PU effect of SNAPHU is similar to that of MCF, and it is also affected by residual noise. Although the above two methods can obtain PU results, due to the influence of pre-filtering, some residual errors will affect the PU results. Figure 4g–i and Figure 4j–l are the results of UKFPU and AUKFMPU. It can be noted that the PU of these two methods is better than the former two methods and the noise in the re-wrapped phase is significantly reduced. The improved method proposed in this paper is better than UKFPU. The residual error after PU is the least. As can be seen from Table 3, the PU results of MCF and SNAPHU are similar. The RMSE are 0.2536 radian and 0.2529 radian, respectively. The PU accuracy of UKFPU and AUKFMPU is 0.1996 radian and 0.1325 radian, respectively, which is clearly better than the former two methods. In particular, the improved method we proposed has a better PU accuracy. From this experiment, it can be seen that the traditional PU method is significantly affected by the pre-filtering. However, excessive or insufficient pre-filtering will produce errors. Pre-filtering has weaker influence on UKFPU and AUKFMPU, especially the proposed improved method, which can obtain good results even in high-noise regions.

### 4.3. GF-3 SAR Experimental Data Results

In this section, two sets of experimental GF-3 SAR data are used for InSAR processing. The real data is the GF-3 SAR data of TangShan, Hebei, China. Table 1 shows some of the parameters of the data. Multi-looks have a certain influence on PU [13]. In this paper, 2 × 2 multi-looks of real data are performed, and the data is filtered by the adaptive data filtering (ADF) with window size of 32 × 32. Then, the datasets are processed by the MCF, SNAPHU, UKFPU, and AUKFMPU methods.

#### 4.3.1. Dataset 1

**Figure 5:**
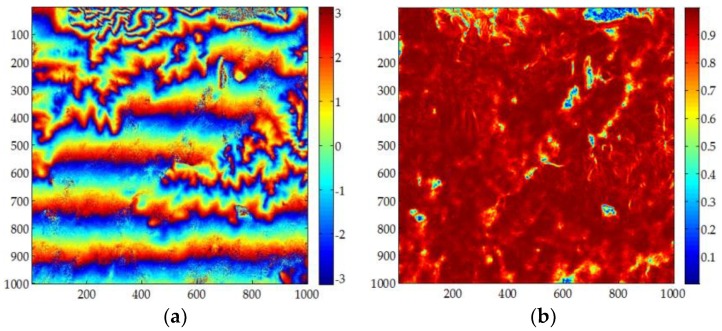
Experimental Dataset 1: (**a**) original interferogram; (**b**) corresponding coherence map.

Figure 5 shows the first set of experimental data. The size of the data is 1000 × 1000 pixels. Figure 5a is the flattened interferometric phase and Figure 5b is the corresponding coherence coefficient map. From Figure 5a,b, it can be seen that, although 2 × 2 multi-looks are performed on the data and is filtered twice; the interferometric phase image still contains noise.

**Figure 6:**
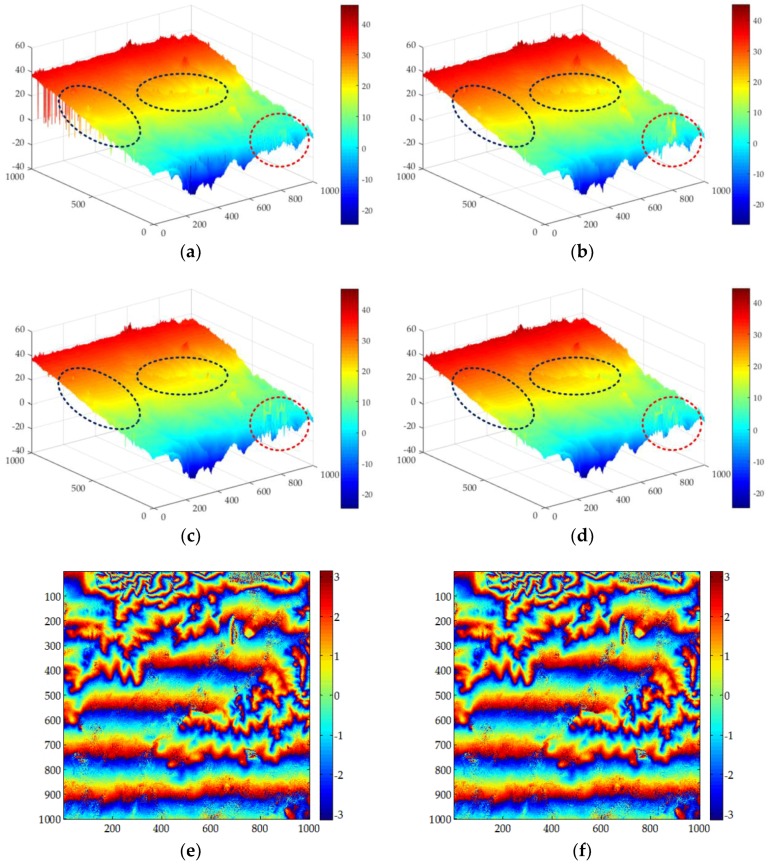

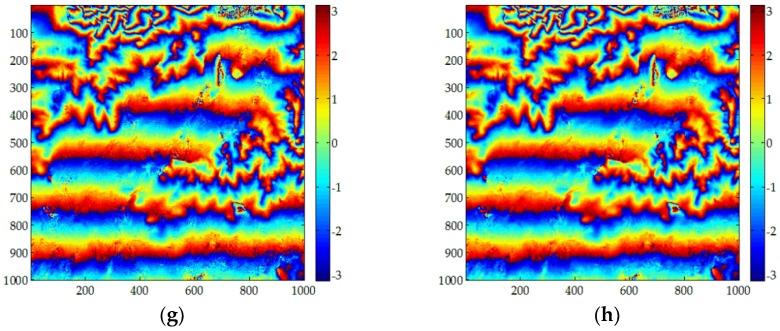
Experimental results for Dataset 1 when the pre-filtering threshold is set at 0.25: (**a**–**d**) are the unwrapped maps of the MCF, SNAPHU, UKFPU, and AUKFMPU methods, respectively; and (**e**–**h**) are the re-wrapped map of the MCF, SNAPHU, UKFPU, and AUKFMPU methods, respectively.

Figure 6 shows the results of the MCF, SNAPHU, UKFPU, and AUKFMPU methods. Figure 6a,e are the PU and re-wrapped results of the MCF method. It can be seen from the results that due to incomplete filtering, some noise still remains in the PU results. This noise has a significant impact on the accuracy of the method. It can also be seen from the results of the re-wrapped that the results of the unwrapping of the MCF are severely affected by the noise. Figure 6b,f are the PU results and re-wrapped results of the SNAPHU method. It can be seen from the figure that there are many noise residuals in the re-wrapped image of the SNAPHU method. Figure 6c,g are the PU and re-wrapped results of the UKFPU method. It can be seen that the UKFPU can perform both PU and filtering simultaneously. It can be seen from the dashed box in Figure 6a–d, both MCF and SNAPHU will produce significant errors. The results of UKFPU are better than those of the former two. However, its robustness still needs further improvement, especially in the high noise region of the red dashed box. The result of AUKFMPU is the best, although there will be some noise residue. It can also be seen from the re-wrapped image that the noise has been reduced. However, there is still some noise residue. Figure 6d,h are the PU and re-wrapped results of the AUKFMPU method. From the results, the improved method proposed in this paper has performed best. 

The number of noise residues has always been one of the main criteria for evaluating the quality of interferometric phase maps. A residue is defined as the pixel whose integral phase along a closure path is not zero. A pixel could be a positive residue when the integral phase is positive and a negative residue when the integral phase is negative. It is in unit of numbers. The more the residues, the poorer the qualities of interferometric phase, and vice versa. As shown in Table 4, it can be seen that the AUKFMPU method yielded the least number of residues, compared to the other methods. This experiment proves that the improved method proposed in this paper is more robust to noise than other methods. Thus, the AUKFMPU method is most suitable for PU of experimental GF-3 SAR data in high-noise and low-coherence regions.

#### 4.3.2. Case 2

**Figure 7:**
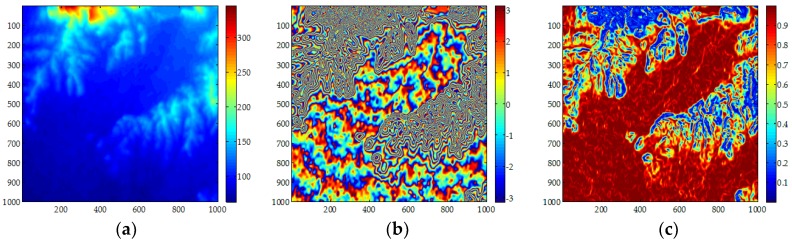
Experimental Dataset 2: (**a**) Shuttle Radar Topography Mission (SRTM DEM); (**b**) original interferogram; (**c**) corresponding coherence map.

Figure 7a shows Shuttle Radar Topography Mission (SRTM) DEM for the second set of data experiments; Figure 7b shows the interferometric phase image for the second set of GF-3 SAR data experiments; while Figure 7c shows the corresponding coherence coefficient image. It can be seen from the Figure 7b that there are some fringe dense regions and the coherence coefficient is also lower in some regions due to the influence of noise. The ADF filter in Gamma software is used to filter the data twice.

**Figure 8:**
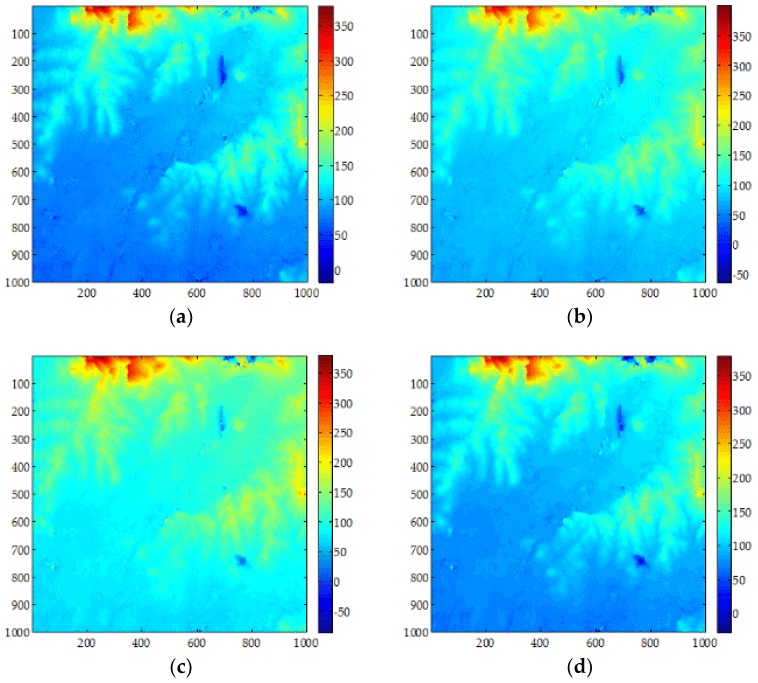

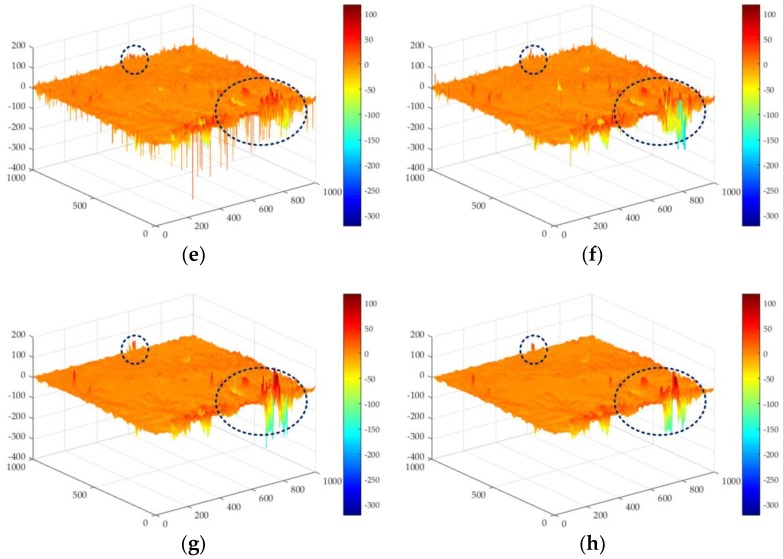
Experimental results for Dataset 2 when the pre-filtering threshold is set at 0.25: (**a**–**d**) are the DEM maps of the MCF, SNAPHU, UKFPU, and AUKFMPU methods, respectively and (**e**–**h**) are the DEM error maps of the MCF, SNAPHU, UKFPU, and AUKFMPU methods, respectively.

Figure 8 and Table 5 show the results from the second set of data processed by the MCF, SNAPHU, UKFPU, and AUKFMPU methods. It can be seen from the figure that, due to the incompleteness of the pre-filtering, the unwrapping results of MCF and SNAPHU are affected by different degrees of noise; the error comparison with SRTM is 5.4141 m and 5.5127 m, respectively. The results of UKFPU are better than MCF and SNAPHU. However, there are still some noise residues that affect the accuracy of the phase. The error comparison with SRTM is 4.9924 m. It can be seen that the result of AUKFMPU is the best. The error comparison with SRTM is 4.7404 m. Figure 8e–h show the results from the different methods and SRTM. Due to the influence of other factors such as water surface, a large DEM error will occur. It can be seen that there are more glitches in the error maps of MCF and SNAHPU. The UKFPU is obviously better than the first two methods. From Figure 8h, we can observe the least glitches in the dashed box, meaning that the AUKFMPU method performs best in processing the gross phase errors. From the comparison of ICESat accuracy, we can see that the minimum error of the MCF method is 0.2063 m, the maximum error is 12.9675 m, and the RMSE is 4.7213 m. Large errors may occur in the water surface regions. The accuracy of the SNAPHU method is similar to that of the MCF. The minimum error of the UKFPU method is 0.0295 m, the maximum error is 11.6766 m, and the RMSE is 4.3774 m. The accuracy of the UKFPU method is significantly better than the first two methods. The minimum error of the AUKFMPU method is 0.0194 m, the maximum error is 11.8693 m, and the RMSE is 4.1020 m. The accuracy of the AUKFMPU method is best. It is further verified that AUKFMPU is more suitable the PU of GF-3 SAR data than other methods.

## 5. Conclusions

In this paper, an improved UKFPU method is proposed. The improved method applies AUKF to PU for the first time and combines AUKF with a circular median filter to perform PU. The proposed method is compared with the MCF, SNAPHU, RPTPU, and UKFPU methods using two sets of simulated data and two sets of experimental GF-3 SAR data. It is shown to yield the greatest accuracy and the greatest robustness to noise compared to the other methods. Especially in regions of high-noise and low-coherence, this method gives better results than other methods. Through the experiment on two sets of simulated data, we can verify that the proposed method can get better results than other methods. The improved method also has better robustness than other methods. Through the experiment on two sets of GF-3 SAR data, it further verified that AUKFMPU can get better results than other methods. The results prove that the proposed method is most suitable for interferometric PU of GF-3 SAR data compared to the other methods considered in this paper, especially in regions of high-noise and low-coherence.

## Figures and Tables

**Table 1:** Experimental image parameters.

Parameter	Value
Wavelength	0.056 m
Image Resolution	1~500 m
Orbital Altitude	755 km
Imaging Bandwidth	10~650 km
Master Image	17 February 2017
Slave Image	18 March 2017

**Table 2:** The root mean square error(RMSE) of minimum cost network flow (MCF), statistical cost network flow (SNAPHU), regularized phase tracking technique (RPTPU), Adaptive unscented kalman filter phase unwrapping (UKFPU), and the proposed method (AUKFMPU) methods for different signal-to-noise ratio (SNR).

SNR (dB)	RMSE (radian)				
	MCF	SNAPHU	RPTPU	UKFPU	AUKFMPU
9	0.2998	0.2998	0.2009	0.1156	0.0839
5	0.5074	0.5078	0.2141	0.1689	0.1182
3	0.6502	0.6501	0.2158	0.2048	0.1702
1	0.8015	0.7961	0.2643	0.2391	0.1999
0.8	0.8576	0.8199	0.2651	0.2451	0.1991
0.5	0.8461	0.8391	0.2589	0.2523	0.1841
0.3	0.8705	0.8548	0.3348	0.2536	0.2069
0.2	0.8705	0.8642	0.2584	0.2605	0.2147

**Table 3:** The RMSE values of different methods.

Method	MCF	SNAPHU	UKFPU	AUKFMPU
**RMSE (radian)**	0.2535	0.2529	0.1996	0.1325

**Table 4:** The residues of different methods.

Method	Original	MCF	SNAPHU	UKFPU	AUKFMPU
**Residues**	4875	4875	4875	917	389

**Table 5:** The accuracy comparison of different methods.

Method	SRTM/m	ICESat/m		
		Min	Max	RMSE
**MCF**	5.4141	0.2063	12.9675	4.7213
**SNAPHU**	5.5127	0.2100	12.4942	4.6859
**UKFPU**	4.9924	0.0295	11.6766	4.3774
**AUKFMPU**	4.7404	0.0194	11.8693	4.1020
